# Over-expression of ASIC1a promotes proliferation via activation of the β-catenin/LEF-TCF axis and is associated with disease outcome in liver cancer

**DOI:** 10.18632/oncotarget.10774

**Published:** 2016-07-22

**Authors:** Cheng Jin, Feng-Lai Yuan, Yuan-Long Gu, Xia Li, Min-Feng Liu, Xiao-Min Shen, Bo Liu, Mao-Qun Zhu

**Affiliations:** ^1^ Department of Hepatobiliary Pancreatic Center, The Third Hospital Affiliated to Nantong University, Wuxi, 214041, Jiangsu, China; ^2^ Department of Research Institute, The Third Hospital Affiliated to Nantong University, Wuxi, 214041, Jiangsu, China

**Keywords:** ASIC1a, β-catenin, CRISPR/CAS9, liver cancer, proliferation

## Abstract

Acid-sensing ion channels 1a (ASIC1a) has been reported to promote migration and invasion in liver cancer. However, the clinical significance and molecular mechanism of ASIC1a in liver cancer remain unknown. In the study, we found that ASIC1a is frequently up-regulated in liver cancer tissues. The over-expression of ASIC1a is associated with advanced clinical stage and poor prognosis. The pro-proliferative of ASIC1a is pH dependent. Knockout of ASIC1a by CRISPR/CAS9 inhibited liver cancer cell proliferation and tumorigenicity *in vitro* and *in vivo* through β-catenin degradation and LEF-TCF inactivation. Our results indicated a potential diagnostic marker and chemotherapeutic target for liver cancer.

## INTRODUCTION

Liver cancer is commonly diagnosed and is identified as the leading causes of cancer death worldwide, with 466100 estimated new cases and 422100 estimated deaths in China and 28410 estimated new cases and 18280 estimated deaths in the United States [1, 2]. Several lines of evidence showed that multiple proteins are deregulated in liver cancer and have been developed as biomarkers or chemotherapy targets such as AFP, PDGF and VEGFR. Nevertheless, diagnostic and therapeutic outcomes remain unsatisfactory [3]. Therefore, more research into the underlying molecular mechanisms of liver cancer tumorigenesis and the identification of de novo biomarkers and specific molecular targets is required urgently.

Acid-sensing ion channels (ASICs) are H^+^-gated cation channels that are widely expressed in mammalian central and peripheral nervous systems and play roles in pain perception, learning and memory, and fear conditioning [4–7]. Among six identified subunits of ASICs, ASIC1a has become a topic of interest in research because of its important biological functions and pathological significance [8]. High rates of glucose metabolism combining with poor perfusion lead to an acid tumor microenvironment in which the pH value is around 6.5 [9]. As an acid-sensing ion channel molecule, ASIC1a is therefore hypothesized to correlate with cancers and has been proved to be involved in glioblastoma and breast cancer proliferation and migration [10–12]. Our group has previously reported that ASIC1a is up-regulated in liver cancer cell and can promote liver cancer cell migration and invasion [13]. However, the overview bio-function, molecular mechanisms and clinical relevance of ASIC1a in liver cancer remain unknown.

β-catenin is an important functional protein in cells. It regulates cell adhesion by binding to cadherins at the intracellular surface. On the other hand, β-catenin mediates downstream signaling transduction by coactive lymphoid enhancer factor/T cell factor (LEF/TCF). LEF/TCF transcriptional activity is principally regulated by nucleus β-catenin level [14, 15]. Several oncogenes such as c-MYC and cyclin D are downstream target genes of LEF/TCF [16]. In cancer cells, β-catenin/LEF/TCF axis is frequently activated and results in an incoercible cell proliferation.

In this study, we found that ASIC1a is over-expressed in 90 liver cancer tissues compared with adjacent normal tissues. ASIC1a over-expression is associated with poor prognosis and clinical features of liver cancer. Loss-of-function study by CRISPR/CAS9 technique indicated that in acid microenvironment ASIC1a can promote liver cancer proliferation *in vitro* and *in vivo* via β-catenin activation and nuclear accumulation. Our findings provide a potential drug target and prognosis biomarker for liver cancer.

## RESULTS

### ASIC1a is up-regulated in liver cancer tissues and cell lines and is associated with poor prognosis

**Figure 1:**
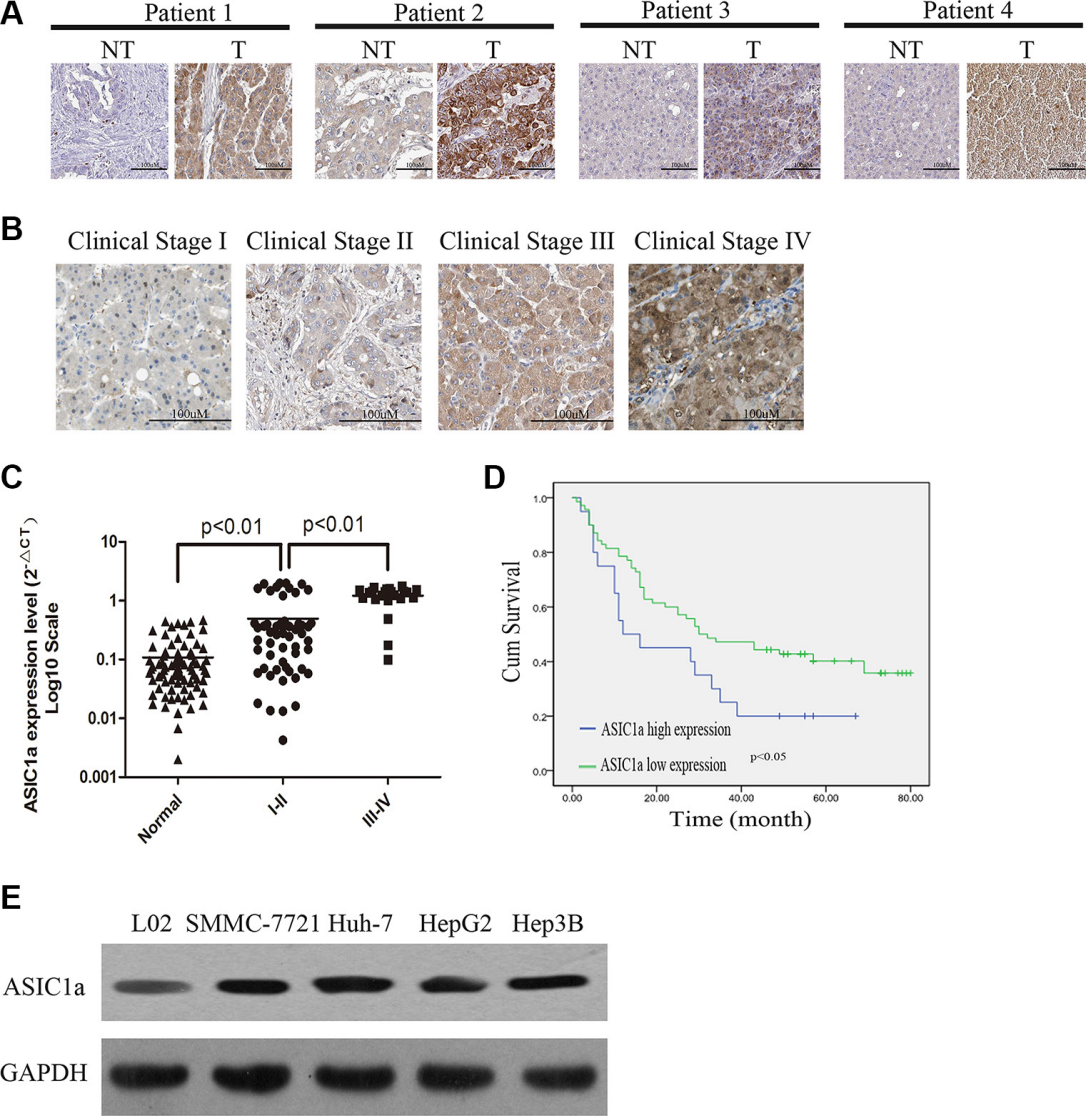
ASIC1a is up-regulated in liver cancer tissues and cell lines and is associated with poor prognosis (**A**) Protein levels of ASIC1a in liver cancer tissues (T) and adjacent normal tissues (NT). (**B**) Protein levels of ASIC1a in different clinical stage. (**C**) *ASIC1a* mRNA levels in 90 liver cancer tissues and adjacent normal tissues *ASIC1a* expression levels were calculated by the *ASIC1a*/*GAPDH* expression ratio (ie, 2^ΔCt^) [34, 35]. The median *ASIC1a* expression level were representing as horizontal line. Liver cancer tissues were divided into two group followed by clinical stage. (**D**) Kaplan-Meier curves with univariate analyses (long-rank) for patients with liver cancer with low ASIC1a expression versus high ASIC1a expression. (**E**) Protein levels of ASIC1a in 4 liver cancer cell lines and normal liver cell line (L02).

We examined ASIC1a expression in 90 pairs of liver cancer tissues and their adjacent normal tissues by immunohistochemical staining. ASIC1a was found significantly up-regulated in tumor tissues and ASIC1a expression in liver cancer increased with advanced clinical stage (Figure 1A and 1B). Moreover, mRNA expression levels of *ASIC1a* were also up-regulated in the same samples and are associated with the clinical stage (Figure 1C). The relationship between ASIC1a protein expression and clinical features including age, gender, tumor size, clinical classification and metastasis state was then analyzed. Fisher's exact analysis revealed that ASIC1a levels were significantly correlated with clinical stage (*p* < 0.001) and Metastasis state (*p* = 0.034) (Table 1). Furthermore, the log-rank test detain 90 liver cancer patients indicated that the overall survival of patients with high levels of ASIC1a was significantly poorer than patients with low levels of ASIC1a protein levels (Figure 1D, *p* < 0.01).

**Table 1:** Clinicopathological characteristics of patient sample and expression of ASIC1a in hepatic carcinoma

Characteristics	ASIC1a		Fisher's Exact Test (*p* value)
	High	Low	
Age (years)			
> 60	8	14	0.22
≤ 60	33	35	
Gender			
Male	38	40	0.11
Female	3	9	
Tumor Size			
≤ 3 cm	8	14	0.46
> 3 cm	33	35	
Clinical stage			
I–II	11	45	< 0.001
III–IV	30	4	
Metastasis			
Yes	31	27	0.034
No	10	22	

Next, we performed western blot to detect ASIC1a expression in liver cancer cell lines SMMC-7721, Huh-7, HepG2, Hep3B and normal liver cell line L02. ASIC1a expression was also higher in liver cancer lines than in L02 (Figure 1E). Of the liver cancer cell lines, SMMC-7721 and Huh-7 showed higher expression. Hence, these two lines were employed for further study.

### Knockout of ASIC1a inhibited liver cancer cells growth *in vitro* and *in vivo*

**Figure 2:**
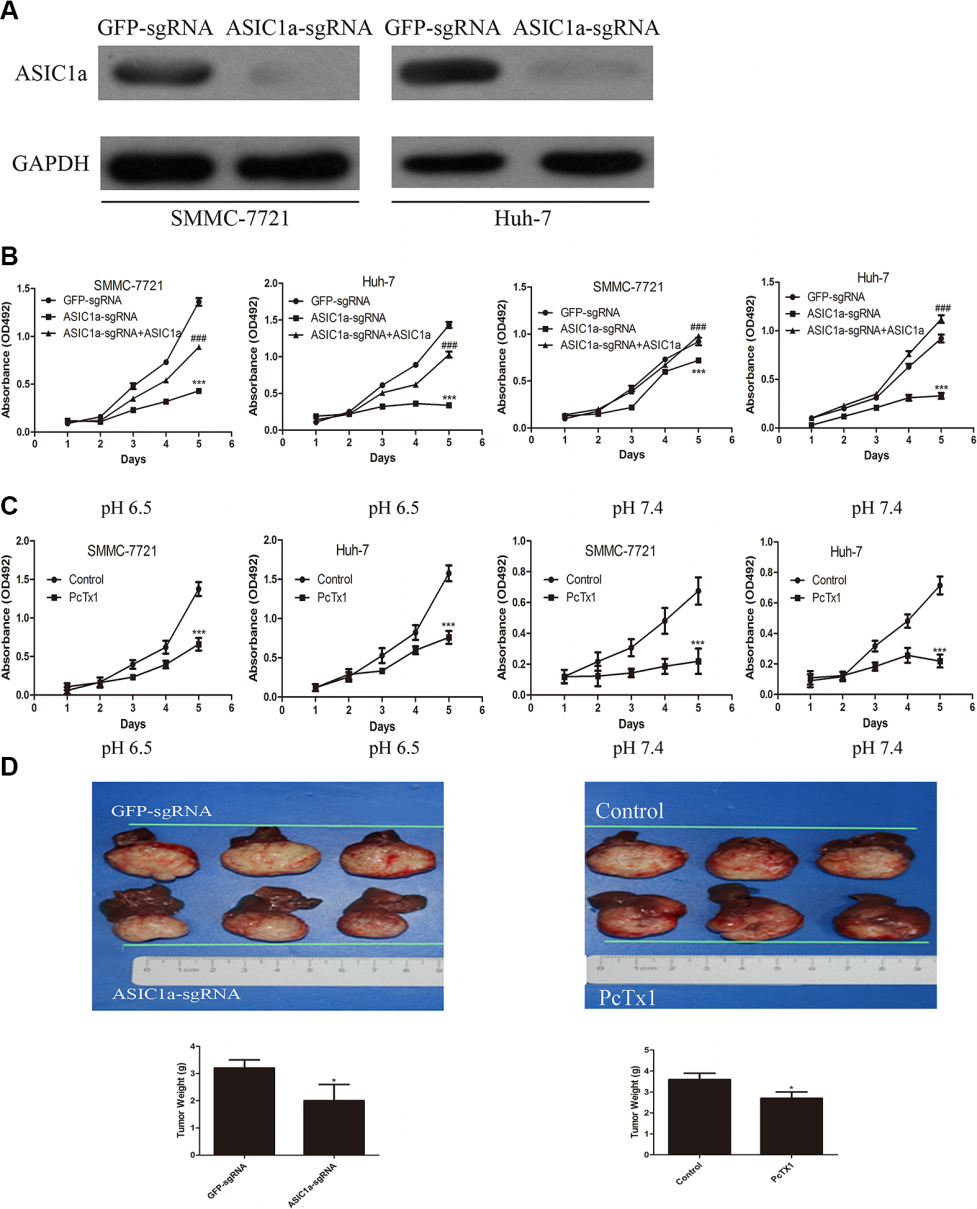
Knockout of ASIC1a inhibited liver cancer cells growth *in vitro* and *in vivo* (**A**) Protein levels of ASIC1a in control and ASIC1a knockout cells. (**B**) MTT assays showed the SMMC-7721 and Huh-7 cells 5 days proliferation ability of ASIC1a-sgRNA, GFP-sgRNA and ASIC1a-sgRNA+ASIC1a lentivirus treated cells in pH 6.5 (left) and pH 7.4 (right). (**C**) SMMC-7721 and Huh-7 were treated with 100 ng/ml PcTx1. (^###^*p* < 0.001 ASIC1a-sgRNA+ASIC1a versus ASIC1a-sgRNA, *t*-test). (**D**) Orthotopic tumor formative ability of ASIC1a-sgRNA, GFP-sgRNA and PcTx1 treated SMMC-7721 cell. *p < 0.05, *p < 0.01, ***p < 0.001, *t*-test.

We have previously reported the pro-metastatic ability of ASIC1a in liver cancer. When the pH of culture medium reduced to 6.5 (similar to the pH value in tumor microenvironment), ASIC1a expression up-regulated simultaneously [13]. Knockdown of ASIC1a by RNA interference inhibited liver cancer cell migration and invasion *in vitro*. In this study, we aimed to investigate the role of ASIC1a in regulating liver cancer cell proliferation. CRISPR/CAS9 technique was performed to knockout ASIC1a in both selected cell lines. As showed in Figure 2A, ASIC1a protein levels dramatically reduced in ASIC1a-sgRNA treated cells when compared with GFP-sgRNA treated cells.

In order to explore the function of ASIC1a on cell growth, SMMC-7721 and Huh-7 cells expressing either ASIC1a-sgRNA lentivirus or GFP-sgRNA lentivirus were seeded in 96-well plates, and cell growth was monitored by MTT every day for 5 days. ASIC1a-sgRNA inhibited SMMC-7721 and Huh-7 liver cancer cell lines proliferation significantly in acid (pH 6.5) and neutral (pH 7.4) microenvironment simultaneously (Figure 2B). Moreover, re-expressed ASIC1a restored the cell proliferation. The inhibitory effect was further confirmed by colony formation assays (Supplementary Figure S1A). Interestingly, we found that when pH reduced to 6.5, clone size of liver cancer cells was larger than those in pH 7.4. ASIC1a-sgRNA exerted a significant inhibitory effect on liver cancer cell colony formation. To further exclude the possibility that the anti-proliferative effect was caused by off-target method, ASIC1a inhibitor PcTx1 was used. As showed in Figure 2C, PcTx1 also significantly inhibited both liver cancer cell proliferations.

Next, we examined whether ASIC1a could promote the tumorigenicity of liver cancer cells *in vivo*. An orthotopicorthotopic xenograft model was performed to mimic the acid microenvironment of liver cancer. Tumors formed from both ASIC1a knockout and PcTx1 treated cells were lighter than tumors formed from GFP-sgRNA and control (PBS treated) tumors (Supplementary Figure 3, *p* < 0.05).

These data demonstrated that knockout of ASIC1a resulted in liver cancer cells growth inhibition *in vitro* and *in vivo*.

### Knockout of ASIC1a induced liver cancer cell apoptosis

**Figure 3:**
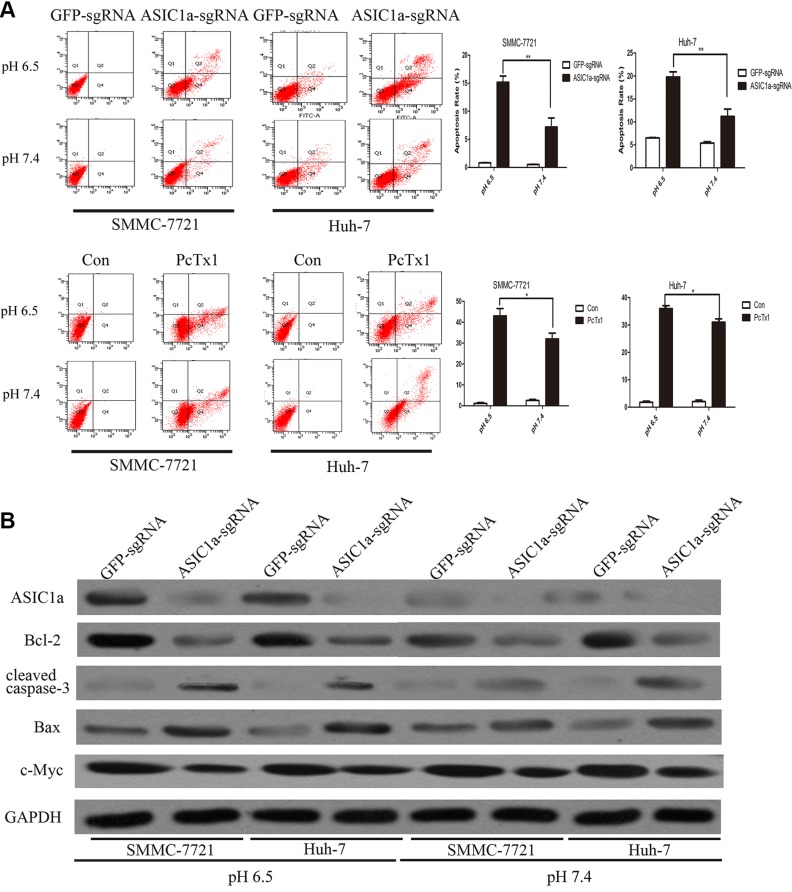
Knockout of ASIC1a induced liver cancer cell apoptosis (**A**) Flow cytometric analysis showed the cell apoptosis rate in ASIC1a-sgRNA, GFP-sgRNA and PcTx1 treated SMMC-7721 and Huh-7 cells in pH 6.5 (top) and pH 7.4 (button). (**B**) Protein levels of ASIC1a, Bcl-2, cleaved caspase-3, Bax, c-Myc in ASIC1a-sgRNA or GFP-sgRNA treated SMMC-7721 and Huh-7 cells in pH 6.5 and pH 7.4. GAPDH was used as a loading control.

A great number of functional genes regulate cell proliferation through apoptosis [17–19]. To gain insights into the mechanism by which ASIC1a regulate liver cancer cell proliferation, we analyzed differences in cell apoptosis following ASIC1a-sgRNA and GFP-sgRNA treatment by fluorescence-activated cell sorting (FACS). As showed in Figure 3A, ASIC1a knockout induced liver cancer cell apoptosis significantly. In addition, the apoptosis rate in pH 6.5 is much greater than in pH 7.4 (*p* < 0.01). Similarly, ASIC1a inhibitor PcTx1 also induced significant apoptosis in both liver cancer cells.

Moreover, we examined the protein expression levels of apoptosis related molecular including Bcl-2, Caspase-3, Bax, c-Myc. The western blotting analysis revealed that the apoptosis induced by ASIC1a knockout be may through intrinsic pathway (Figure 3B).

### Knockout of ASIC1a induced liver cancer cell cycle arrest through LEF-TCF activity inhibition

**Figure 4:**
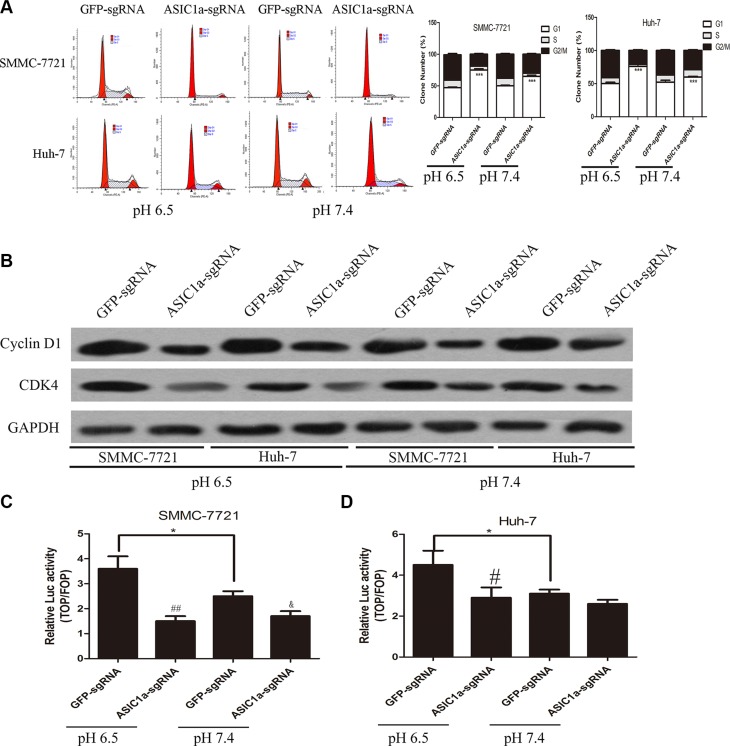
Knockout of ASIC1a induced liver cancer cell cycle arrest through LEF-TCF activity inhibition (**A**) Flow cytometric analysis showed the cell cycle arrest in ASIC1a-sgRNA or GFP-sgRNA treated SMMC-7721 and Huh-7 cells in pH 6.5 (left) and pH 7.4 (right). (**B**) Protein levels of Cyclin D1 and CDK4 in ASIC1a-sgRNA or GFP-sgRNA treated SMMC-7721 and Huh-7 cells in pH 6.5 and pH 7.4. GAPDH was used as a loading control. LEF-TCF assay showed the LEF-TCF activity in ASIC1a-sgRNA or GFP-sgRNA treated (**C**) SMMC-7721 and (**D**) Huh-7 cells in pH 6.5 and pH 7.4. *p < 0.05, **p < 0.01, ***p < 0.001, #*p* < 0.05, ##*p* < 0.01 (ASIC1a-sgRNA versus GFP-sgRNA), *t*-test.

Cell cycle arresting is one of the signals that can trig apoptosis [20]. We then we analyzed differences in cell cycle distributions following ASIC1a-sgRNA and GFP-sgRNA treated liver cancer cells by fluorescence-activated cell sorting (FACS). As showed in Figure 4A, G1/S transition was arrested after ASIC1a knockout and the arresting effect was much greater in pH 6.5 than in pH 7.4 in SMMC-7721 and Huh-7 liver cancer cells.

G1/S transition was regulated by several genes. We then analyzed the expression levels of G1 dependent modulators Cyclin D1 and CDK4 western blotting. Figure 4B confirmed that ASIC1a knockout can induce liver cancer cells arresting in G1 phase.

Interestingly, we found that apoptosis and cell cycle related gene that we examined, c-Myc and Cyclin D1, were also Wnt/β-catenin/LEF-TCF target genes. Moreover, other Wnt/β-catenin/LEF-TCF target genes Osx and Runx2 mRNA level were also decreased after ASIC1a knockout (Supplementary Figure S1B). LEF-TCF reporter assay were then employed to determine whether β-catenin/LEF-TCF is downstream of ASIC1a mediated liver cancer cell proliferation inhibition. Figure 4C and 4D indicated that LEF-TCF activity elevated in acid medium. ASIC1a knockout resulted in a notable decrease in LEF-TCF activity in both liver cancer cell lines.

These results indicated that ASIC1a knockout may inhibit liver cancer cell proliferation through β-catenin/LEF-TCF axis.

### ASIC1a knockout suppressed activation of β-catenin/LEF-TCF axis

**Figure 5:**
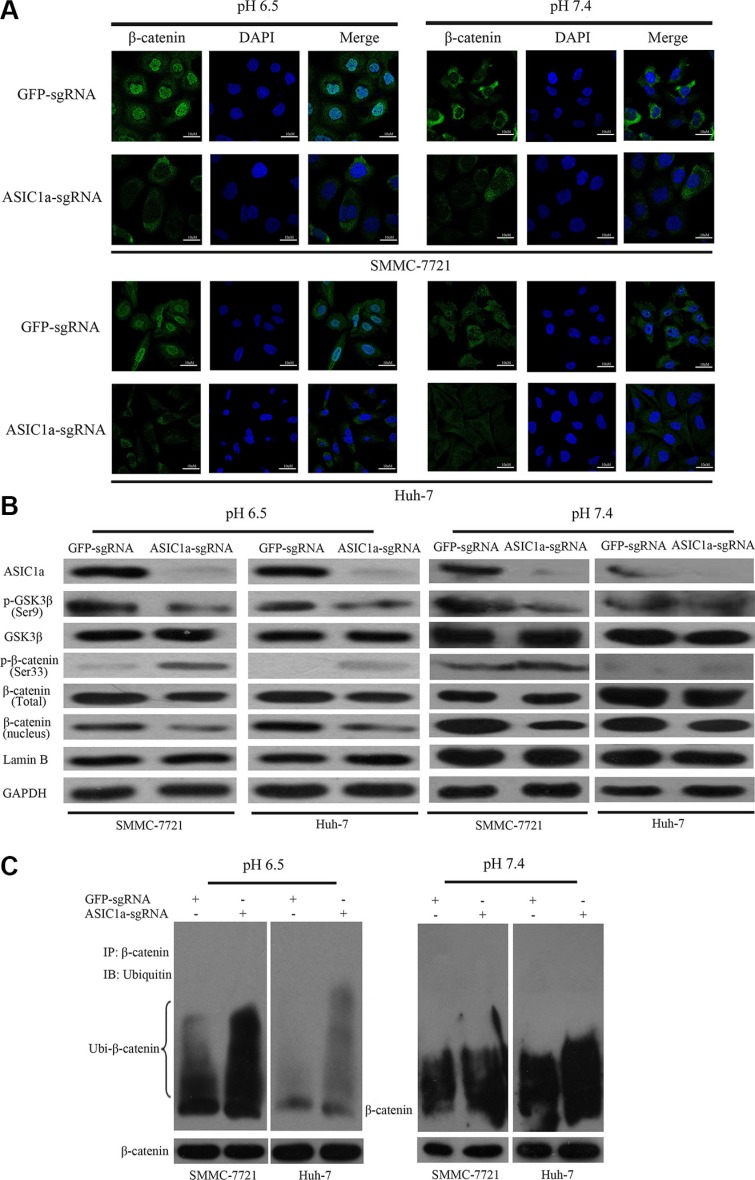
ASIC1a knockout suppressed activation of β-catenin/LEF-TCF axis (**A**) The translocation of β-catenin from the cytoplasm to the nucleus was detected by immunofluorescence staining following ASIC1a-sgRNA or GFP-sgRNA treated SMMC-7721 and Huh-7 cells in pH 6.5 and pH 7.4. (**B**) Protein levels of ASIC1a, GSK3β, p-GSK3β (Ser 9), β-catenin, p-β-catenin (Ser 33). Lamin B and GAPDH were used as loading controls. (**C**) Whole cell extracts were subjected to immunoprecipitation with V5 antibody and the blots were probed with HA antibody. We also probed the same blots for total β-catenin to show relatively equal immunoprecipitation of total β-catenin.

Mounting evidences indicate that β-catenin/LEF-TCF is one of the key signaling pathways in modulating cell proliferation, migration and adhesion [21, 22]. To explore whether there exist relativity between ASIC1a and β-catenin, we then observed the cellular localization of β-catenin in ASIC1a-sgRNA or GFP-sgRNA treated cells in different pH. The Immunofluorescence picture showed that β-catenin accumulated in nuclear when pH 6.5. ASIC1a knockout not only reduced total β-catenin expression levels but also significantly inhibited the nuclear accumulation of β-catenin in both liver cancer cells (Figure 5A).

Next, we examined β-catenin regulator GSK3β and its phosphorylated form. Figure 5B, Supplementary Figure 2 and Figure 5C showed that when cells were treated with ASIC1a-sgRNA, GSK3β was activated and β-catenin was phosphorylated. β-catenin was then degraded through an ubiquitination dependent pathway. Similarly, the signal transduction is more significant in pH 6.5 environment. As a result, the nuclear accumulation of β-catenin was inhibited and the downstream LEF-TCF activity was inactivated.

These results indicated that in acid microenvironment, ASIC1a may protect β-catenin from phosphorylation and ubiquitination; help β-catenin nuclear accumulation and then promote liver cancer cell proliferation.

## DISCUSSION

Taken together, the findings of the current study showed that both mRNA and protein levels of ASIC1a are over-expressed in liver cancer tissues compared with adjacent normal tissues. ASIC1a over-expression is involved in advanced liver cancer clinical stage and poor prognosis. Knockout of ASIC1a induced G1/S phase arresting; initiated cell apoptosis and then inhibited the cell proliferation of liver cancer cells. We also showed that ASIC1a promote liver cancer proliferation through β-catenin protection and nuclear accumulation. To our knowledge, this study offers new insights and strong evidences that ASIC1a is a potential oncogene in liver cancer.

In this study, we first explored the clinical relevance between liver cancer and ASIC1a expression level. Both mRNA and protein levels were up-regulated in 90 liver cancer patient samples that we collected. The over-expression of ASIC1a is significantly correlated with advanced clinical stage and poor prognosis, which indicated that ASIC1a may be used as liver cancer diagnostic and prognostic biomarker.

ASIC1a has been reported to mediate neuronal death and tonic-clonic seizures in pH 6.0 or even lower [23, 24]. Nevertheless, the pH in tumor microenvironment is around 6.5. Experimental data from cancer cells that cultured in natural medium may not reflect the fact. Hence, in this study, we used pH 6.5 to mimic the tumor microenvironment. pH 7.4 culture medium was also used as a control condition. Our data showed that ASIC1a exert a stronger regulating effect in pH 6.5 environments. Furthermore, nude mice experiments data indicated that ASIC1a knockout and inhibition were able to impair tumor formation *in vivo* respectively. Our data are consistent with a loss of channel activity but that whether or not an ASIC1 channel or a channel containing ASIC1 as a component in liver cell remains to be determined. Further evidences that are reflective of channel activity such as patch-clamp data is necessary in the following research [25].

**Figure 6:**
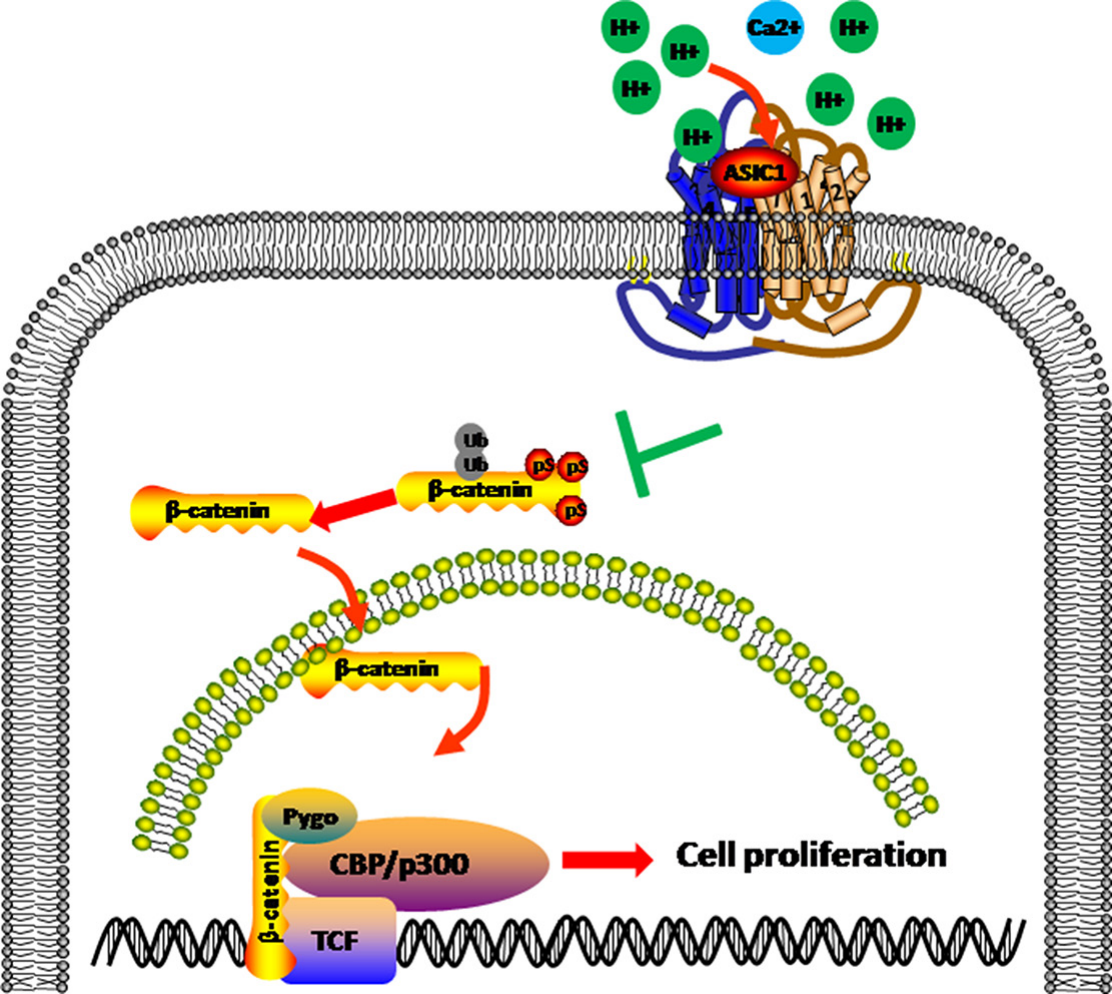
ASIC1a induce β-catenin nuclear translocation and accumulation In pH 6.5 environment, ASIC1a decrease the Ser33 phosphorylation of β-catenin and help β-catenin accumulation and nuclear translocation by avoiding ubiquitination. The nucleus β-catenin combined with LEF/TCF and promotes target genes transcription to help liver cancer proliferation.

Interestingly, we found that β-catenin/LEF-TCF target genes were significantly reduced followed by ASIC1a knockout. Tumor microenvironment acidification results in H^+^ accumulated. H^+^ competes the Ca^2+^ binding site on ASIC1a; depolarize the cell membrane; promote the Ca^2+^ internal flow [26, 27]. On the other hand, β-catenin phosphorylation and ubiquitination was Ca^2+^dependent [28]. We then hypothesized that ASIC1a may exert function through β-catenin/LEF-TCF signal. ASIC1a knockout resulted in GSK3β activation and β-catenin phosphorylation and ubiquitination. Ubiquitin targeted β-catenin was then degraded and the nuclear accumulation of β-catenin decreased. Eventually, LEF-TCF activity was deprived and downstream oncogenes expression levels decreased (Figure 6).

In conclusion, our results suggest that ASIC1a is critical for liver cancer cell proliferation and its deregulation is frequently linked to liver cancer.. Full understanding of the precise role of ASIC1a in human cancer may provide the opportunity to develop a novel therapeutic strategy by suppressing expression of ASIC1a in liver cancer cells. Furthermore, ASIC1a is also a potential diagnostic and prognostic biomarker for liver cancer.

## MATERIALS AND METHODS

### Cell lines

Liver cancer cell lines SMMC-7721, Huh-7, HepG2, Hep3B and normal liver cell L02 were obtained from Cell Bank of Type Culture Collection of Chinese Academy of Sciences (Shanghai, China) and cultured in Dulbecco's Modified Eagle's Medium (Gibco) supplemented with 10% FBS (HyClone). Dilute hydrochloric acid and sodium bicarbonate were added to the complete medium to adjust pH and then filter sterilized. The medium was changed every 2 days, and cells were cultured in two different pHs, including 6.5, and 7.4. Cells were passaged and digested with 0.25% trypsin for cell fusion as a single layer.

### Reagents

The antibodies used: anti-ASIC1a, anti-Bcl-2, anti-cleaved-caspase3, anti-Bax, anti-c-Myc, anti-Cyclin D, anti-CDK4, anti-GSK3β, anti-p-GSK3β (Ser9), anti-β-catenin, anti-p-β-catenin (Ser33) were from Abcam (Cambridge, UK). Anti-GAPDH, anti-HA and anti-Lamin B were from Santa Cruz Biotechnique (Santa Cruz, USA). Dual luciferase reporter assay system was from Promega Corporation (Wisconsin, USA). Reporter constructs were generated by incorporating 8X lymphocyte enhancer-binding factor-T cell factor (LEF-TCF) consensus binding sites into the pGreenFire1 (pGF1) vector containing eGFP-T2A-lucifersase as the reporter (System Biosciences) [29]. cDNAs were cloned into mammalian expression vectors pCDNA6-CMV-V5/His (Invitrogen) or a modified pCDH1-EF1 vector (System Biosciences). We obtained from Addgene the pcDNA3-HA-Ub construct (no. 18712) [30]. Lenti-cas9 and Lenti-sgRNA were purchased from Genechem (Shanghai, CHN). Cells were firstly infected with lenti-cas9 and selected by puromycin. The stable sub-lines were then infected with lenti-sgRNA to specifically knockout target genes. The sgRNA used were sg-GFP: 5′- GGTGAACCGCATCGAGCTGA-3′; sg-ASIC1a: 5′- GACGAGACGTCCTTCGAAGC-3′. All other chemicals were from Sigma–Aldrich Corporation (San Luis, USA) unless otherwise stated.

ASIC1α-specific inhibitor PcTx1 was purchased from Abcam (ab120483, Cambridge, MA, USA).

### CRISPR/CAS9 knockout

Liver cancer cells were transfected with lenti-cas9 virus. Cells transfected with lenti-cas9 gain puromycin resistance. All transfected cells were selected by 5 uM puromycin for 5 days. The stable puromycin resistant sub-lines were then infected with ASIC1a specific sgRNA lentivirus for another 7 days. Lenti-sgRNA vector contains EGFP for infection efficiency evaluation. Lenti-sgRNA trasfected cells were then used for further study. The knockout effect was tested by western blot.

### Patients and information

90 liver cancer samples and adjacent normal liver tissues were collected from patients were diagnosed with liver cancer from 2005 to 2015 at the third hospital affiliated to Nantong University. All tissues were divided equally for paraffin embedded and liquid nitrogen conservation respectively.

Tumor stages were determined according to the TNM system of the American Joint Committee on Cancer [31]. The histological grade of each tumor was determined based on the Edmondson-Steiner grading system [32].

The experiments were undertaken with the understanding and written consent of each subject; and the study methodologies conformed to the standards set by the Declaration of Helsinki. Patient information is in Table 1.

### Animals

Nude mice (BALB/C, male, about 4–6 weeks of age) were employed and raised under SPF conditions. All *in vivo* experiments were performed according to our institutions’ guidelines for the use of laboratory animals and were approved by the Institutional Animal Care and Use Committee of the third hospital affiliated to Nantong University.

For orthotopic implantation, 5*10^6^ GFP-sgRNA and ASIC1a-sgRNA lentivirus treated CAS9-SMMC-7721 cells suspended in 50 ul PBS were injected into the liver lobe of nude mice. On day 28, animals were euthanized and tumors were excised and weighted.

For PcTx treatment, PcTx (10 ng/kg) was tail vein injection every 3 days.

### RNA extraction and quantitative real-time PCR

Total RNA was extracted using TRIzol^®^ Reagent (Invitrogen, CA, USA). To quantitate the gene expression, reverse transcription was performed with a specific stem-loop real-time PCR miRNA kit (RiboBio, Guangzhou, China). Quantitative real-time PCR (qPCR) was performed using the SYBR Green qPCR system (Takara, Dalian, CHN) on an Applied Biosystems 7900HT real-time PCR system. GAPDH was used as an internal control. All samples were normalized to internal controls.

### Western blot analysis

Whole cell lysates were prepared in buffer containing 0.5% Triton X-100, 120 mM NaCl, 50 mM Tris (pH 8.0), 2 mM ethylenediaminetetraacetic acid (EDTA), 1 mM Na2-VO4 and 1:300 protease Inhibitor cocktail (P8340; Sigma-Aldrich). Proteins were resolved by sodium dodecyl sulfate (SDS)–polyacrylamide gel electrophoresis (PAGE). The membranes were incubated with antibodies (Sigma, St Louis, MO, USA) followed by horseradish peroxidase (HRP)-conjugated IgG (Abcam), and the bands were detected using the Supersignal West Pico ECL chemiluminescence kit (Pierce) and Kodak X-ray film (Eastman Kodak Co, NY, USA).

### Immunohistochemistry

Formalin-fixed, paraffin-embedded tissue sections (5 μm) were deparaffinized in xylene and rehydrated with gradient concentrations of ethanol. Endogenous peroxidase activity was blocked (0.35% H_2_O_2_ in PBS buffer), antigens were retrieved by microwaving (350 W), and nonspecific binding was blocked by 1% bovine serum albumin in PBS buffer. Sections were stained with ASIC1a antibody (diluted 1:500; Abcam, ab122396) and visualized with secondary antibody (Envision, DakoCytomation). Slides were then incubated with 3, 3′-diaminobenzidine chromogen (DakoCytomation), counterstained in Meyer's hematoxylin, and mounted with Aquatex (Merck, Darmstadt, Germany). Samples were scored using the IHS value as described by Soslow [33]. IHS = A × B, where A is the percentage of positive cells and B is the positive cell staining intensity grade. B was scored from 0 to 3 as follows: 0, negative; 1, low positive; 2, medium; and 3, strong positive. Score <= 1 indicates low expression. Score > 1 indicates high expression.

### MTT assay

Cells were infected with ASIC1a-sgRNA or GFP-sgRNA lentivirus over night and then plated in six-well plates at density of 105 cells/well. Cell growth was determined using the 3-(4,5-methylthiazol-2-yl)-2,5-diphenyltetrazolium bromide (MTT) colorimetric growth assay for 5 days. Each day, cell growth was determined by adding MTT solution (50 μg/well) for 4 h. Cellular MTT was solubilised with acidic isopropanol and optical density was measured at 570 nm. The doubling time was calculated for the exponential growth phase. All experiments were performed 3 times in triplicates. The viability of the cells was assessed from three replicates in three independent experiments by the MTT.

### Colony formation assay

Cells infected with ASIC1a-sgRNA lentivirus or GFP-sgRNA lentivirus was seeded in 6-well plates at a density of 800 per well and cultured at 37°C for 14 days. Medium was replaced every 2 to 3 days. Cells were washed twice with PBS, fixed with 4% paraformaldehyde, stained with Giemsa for 10 minutes and washed 3 times with double distilled H_2_O. Colonies were photographed and counted under a microscope (Leica DM IL; Leica Microsystems).

### Cell apoptosis analysis

Cell apoptosis was assayed by staining with Annexin V-FITC (ebioscience, 88–8007) and PI following manufacturer's instructions and detected by a flow cytometer (FACSCalibur, Becton Dickinson).

### Cell cycle analysis

Cells infected with ASIC1a-sgRNA lentivirus or GFP-sgRNA lentivirus were collected, washed twice with ice-cold phosphatebuffered saline (PBS), and fixed with 70% ice-cold ethanol. After fixation overnight and subsequent rehydration in PBS for 30 min at 4°C, the samples were stained for 30 min in dark with 50 g/ml propidium iodide (Sigma-Aldrich, P4170) containing 125 U/ml protease-free RNase, and then analyzed using a flow cytometer (FACSCalibur, Becton Dickinson). Cell cycle analysis was carried out using ModFit 2.0 software (Becton Dickinson).

### Immunofluorescence

Cells were fixed in 4% paraformaldehyde, permeabilized with 0.25% Triton and incubated with primary antibodies at 4°C overnight, followed by incubation with rhodamine-conjugated goat antibodies against rabbit IgG (abcam, Cambridge, UK). Nuclei were counterstained with DAPI, and then the coverslips were imaged via confocal laser scanning microscope (Olympus FV1000, Olympus, Center Valley, USA).

### LEF-TCF reporter assay

Cells were transfected using Lipofectamine2000 (Invitrogen) following the manufacturer's general guidelines. For the Luciferase assays, cells were lysed in 1× passive lysis buffer and the SteadyGlo reagent was used as the substrate (Promega).

### Cell-based ubiquitination assays

Cells were transfected with sgRNAs, V5-tagged β-catenin and HA-ubiquitin using Lipofectamine2000. Whole cell extracts (approximately 500–750 ug) were incubated with 1 to 2 μg anti-V5 antibody and incubated at 4°C with rocking overnight. Twenty to 40 μL of protein G plus agarose was added and samples were incubated for an additional hour at 4°C with rocking. The beads were then washed 4 times with lysis buffer (0.5% Triton X-100, 120 mM NaCl, 50 mM Tris [pH 8.0], 2 mM EDTA). Proteins were resolved with SDS-PAGE and ubiquitinated β-catenin was detected with an anti-HA antibody.

### Statistical analyses

Averages are from independent experiments with SD or SEM used to display variability. The Student *t* test when performed as indicated is with a 2-tailed test. Survival curves were calculated using Kaplan–Meier and log-rank tests. The Mann-Whitney test was used to analyze the relationship between ASIC1a mRNA in different tissues. *p* < 0.05 was considered to be statistically significant.

## SUPPLEMENTARY MATERIALS FIGURES


