# Supplementary material for: Over-expression of ASIC1a promotes proliferation via activation of the β-catenin/LEF-TCF axis and is associated with disease outcome in liver cancer
Source: Oncotarget. 2016 Jul 22;8(16):25977–88. doi: 10.18632/oncotarget.10774 (PMC5432231; doi:10.18632/oncotarget.10774)
Supplement: Supplementary file 1 [file oncotarget-08-25977-s001.pdf]

Over-expression of ASIC1a promotes proliferation via activation of the β-catenin/LEF-TCF axis and is associated with disease outcome in liver cancer

Supplementary Materials

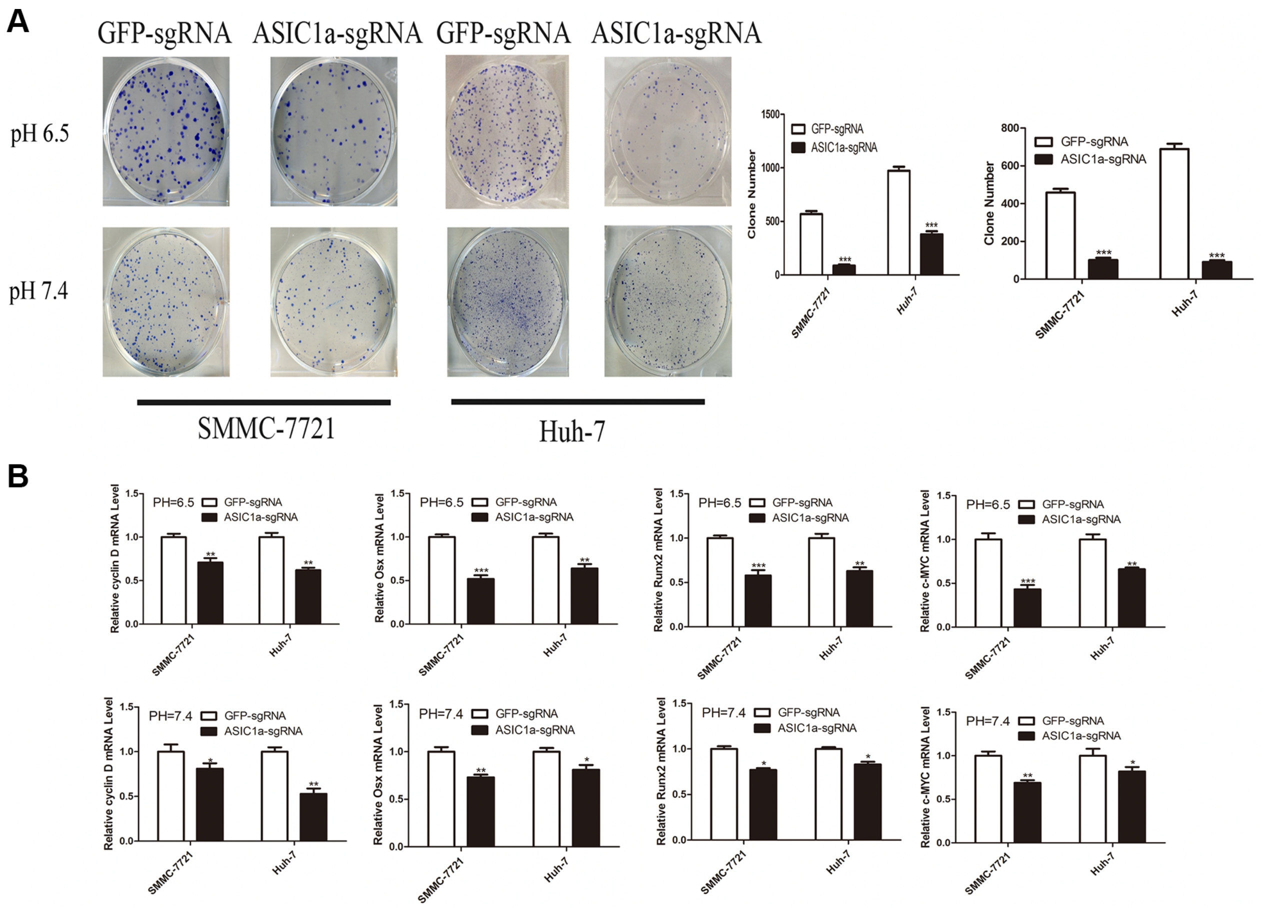

Supplementary Figure S1: (**A**) Colony formation assay showed the colony formative ability of ASIC1a-sgRNA or GFP-sgRNA treated SMMC-7721 and Huh-7 in 14 days in pH 6.5 (top) and pH 7.4 (button). (**B**) LEF-TCF target genes cyclin D, Osx, Runx2 and c-MYC mRNA levels in GFP-sgRNA and ASIC1a-sgRNA treated SMMC-7721 and Huh-7 cell.

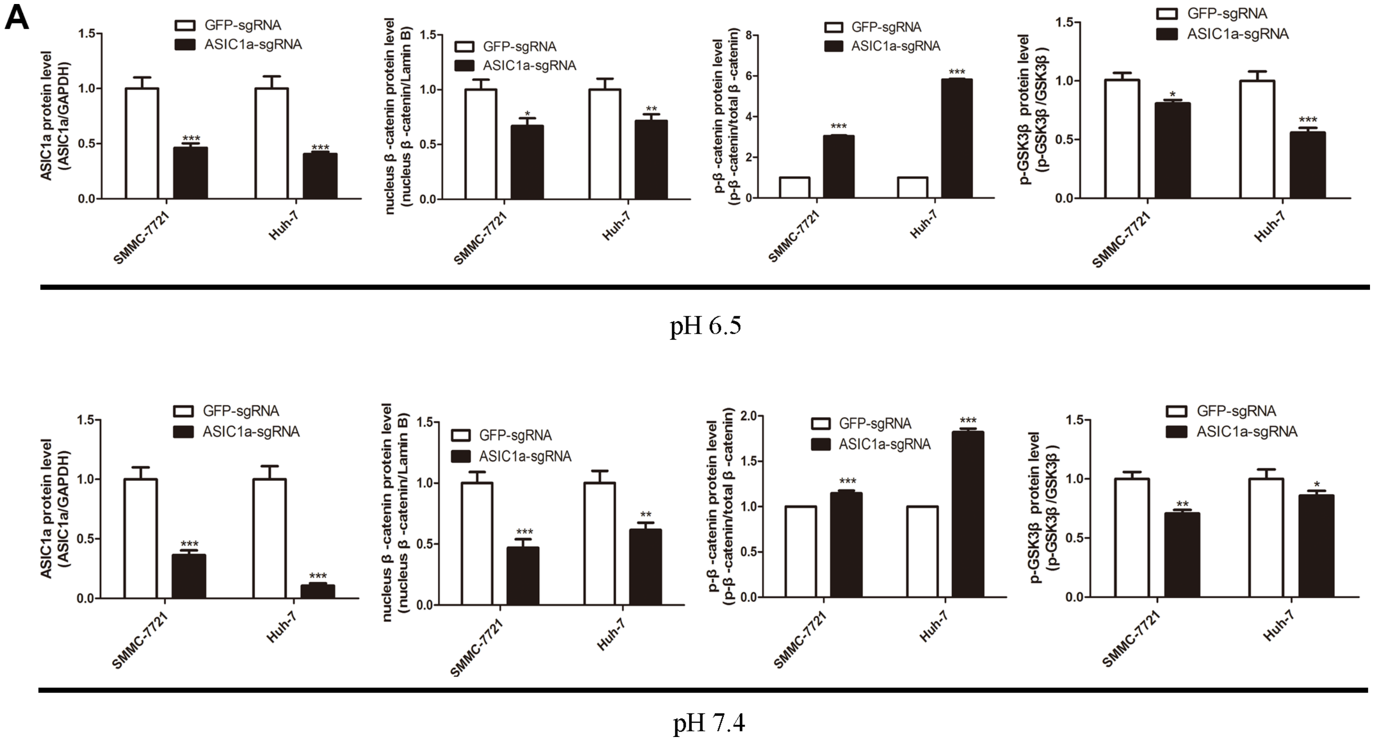

Supplementary Figure S2: (**A**) Densitometry changes between the protein levels of ASIC1a, nucleus β-catenin, p-β-catenin and p-GSK-3β in GFP-sgRNA and ASIC1a-sgRNA treated SMMC-7721 and Huh-7 cells.

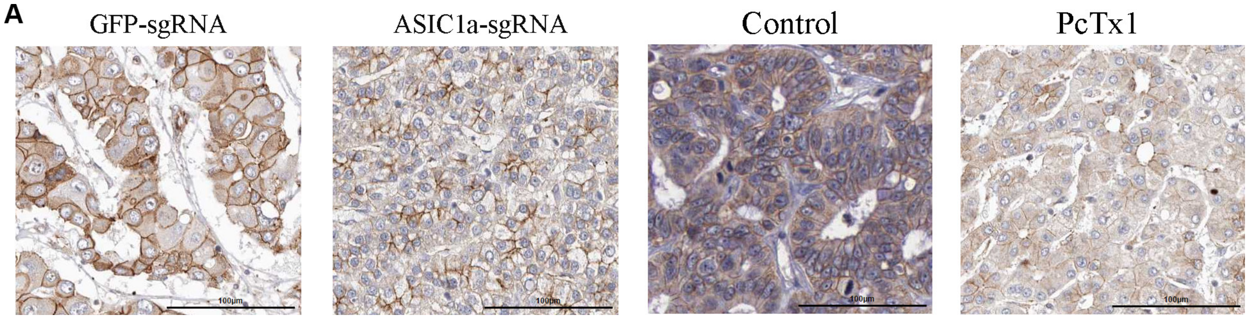

Supplementary Figure S3: (**A**) Localization of β-catenin in tumor tissues from nude mice treated with GFP-sgRNA, ASIC1a-sgRNA and PxTx1.
